# Epithelial Membrane Protein-3 and Chitinase-3-like Protein-1 as New Prognostic Predictors of Glioma, a Two-Gene Study

**DOI:** 10.3390/curroncol30100629

**Published:** 2023-09-23

**Authors:** Kecheng Shen, Jiandong Zhu, Shijie Zhou, Xin Jin, Weiwei Zhai, Liang Sun, Jiang Wu, Zhengquan Yu

**Affiliations:** Department of Neurosurgery, The First Affiliated Hospital of Soochow University, 188 Shizi Street, Suzhou 215006, China; kecheng.shen@neurosci.com.cn (K.S.); chanton.zhu@neurosci.com.cn (J.Z.); shijie.zhou@neurosci.com.cn (S.Z.); xin.jin@neurosci.com.cn (X.J.); pie_2000@neurosci.com.cn (W.Z.); pie_2007@neurosci.com.cn (L.S.)

**Keywords:** EMP3, CHI3L1, glioma, glioblastoma, gene, prognostic

## Abstract

Background: Glioblastoma multiforme is the most common primary intracranial tumor, with a high degree of malignancy, poor therapeutic effect, and poor prognosis. According to previous studies, CHI3L1 and EMP3 are two independent tumor predictors that are of great significance for the prognostic prediction of other tumors, and their expression levels may be related to the prognosis of glioma patients. Methods: using Oncomine, Gene Expression Profiling Interactive Analysis (GEPIA), the Chinese Glioma Genome Atlas (CGGA), cBioPortal, LinkedOmics, and other databases, 693 glioma patients were screened to analyze the relationship between EMP3 and CHI3L1 expression and prognosis in glioma patients. Results: low-grade glioma patients with a low expression of EMP3/CHI3L1 had a better prognosis, and the combination of EMP3/CHI3L1 is a new predictor for glioma patients. Conclusion: We used the TCGA and CGGA databases to analyze the effect of EMP3 and CHI3L1 expression on the prognosis of glioma patients and their correlation with gene expression using bioinformation analysis. The results showed that low-grade glioma patients with a low expression of EMP3 and CHI3L1 had a better prognosis, and EMP3 and CHI3L1 co-expression genes were correlated. The combination of these two factors could be a new prognostic index for glioma patients.

## 1. Introduction

Glioma is the most common primary intracranial tumor, accounting for 70% of all central nervous system intracranial tumors, and the most common type is glioblastoma multiforme (GBM) [1,2,3]. Histologically, GBM is characterized by diffuse infiltration of tumor cells, nuclear atypia, a high mitotic index, microvascular proliferation, and necrosis [4,5]. Although there are various methods for treating glioma, including surgery, radiotherapy, chemotherapy, targeted therapy, and the tumor treatment field (TTF), the prognosis for patients is poor. The five-year survival rate for glioma is approximately 36% [1,2,3], and the five-year survival rate for GBM is less than 5%. The median survival time is approximately 12–18 months [6,7]. Treatment effectiveness is influenced by tumor heterogeneity as well as genetic and epigenetic factors. Anke Zhang et al. argued that aggressive treatment can impair a patient’s quality of life and produce harmful adverse effects. Therefore, understanding the mechanisms underlying tumor progression is essential for disease management and prognosis prediction [8]. Gerber et al. found that reduced mRNA expression levels of CHI3L1, EMP3, and other genes are associated with prolonged survival in glioma patients [9]. This is consistent with the results of Jovčevska I [10], but they did not investigate the relationship between EMP3 and CHI3L1.

Epithelial membrane protein-3 (EMP3) is a 163-amino acid four-transmembrane protein (~18 kDa) encoded by the EMP3 gene on the human chromosome 19q13.3 [11,12,13]. EMP3 belongs to the peripheral myelin protein 22 (PMP22)/dense protein superfamily, which is widely expressed and significantly upregulated in some tissue types [11,14]. In terms of subcellular localization, EMP3 is expected to be present in the plasma membranes and cytoplasmic vesicles [15]. In recent years, abnormal expression of EMP3 has been observed in many cancers, and many studies have focused on EMP3 in tumor progression and malignant transformation. EMP3 promotes tumor growth and metastasis through the PI3K/AKT pathway, and is highly expressed in upper urinary tract urothelial carcinoma (UTUC) and hepatocellular carcinoma (HCC) [16,17]. Its enrichment in monocytes was further confirmed by single-cell RNA sequencing (scRNA-seq), which showed a high expression of EMP3 in macrophages and specialized macrophages, such as Kupffer cells in the liver and Hofbauer cells in the placenta [18]. The EMP3 expression appears to be relatively low in the adult brain compared to the blood and other tissues and has little regional specificity [14,18]. This was further confirmed by the recently published snRNA-seq data set for the adult brain, which showed that in addition to endothelial cells, astrocytes, and microglia, EMP3 expression is low in most cell types in the cortex [19]. EMP3 expression is significantly higher in glioma cells than in non-neoplastic white matter [20,21]. A recent study showed that EMP3 has oncogenic properties in high-grade glioma (HGG), and its overexpression may also predict poor clinical prognosis in GBM [20,22]. According to several studies [20,22,23], EMP3 is frequently overexpressed in IDH-wt GBM.

Chitinase-3-like protein-1 (CHI3L1) is a non-enzymatic chitinase-like protein (CLP) that belongs to the glycoside hydrolase family. It binds chitin, heparin, and hyaluronic acid and is regulated by extracellular matrix changes, cytokines, growth factors, drugs, and stress. CHI3L1 is closely associated with asthma, arthritis, sepsis, diabetes, liver fibrosis, and coronary artery disease [24]. CHI3L1 is produced by various cells and overexpressed in many human cancer types and animal tumor models, such as oligodendroglioma and glioblastoma [25]. CHI3L1 plays a crucial role in preventing pathogen-, antigen-, and oxidant-induced injury responses, inflammation, and tissue repair and remodeling by regulating a variety of fundamental biological processes, including oxidative damage, apoptosis, cell death, inflammasome activation, and Th1/Th2 inflammation [24]. In glioblastoma, CHI3L1 regulates tumorigenesis by disrupting the guidance pathway [26]. In addition, CHI3L1 protects cancer cells from apoptosis by remodeling the extracellular matrix (ECM), thereby creating a suitable substrate for tumor growth and progression [27]. Patients with overexpressed CHI3L1 tumor cells have a high rate of tumor metastasis and a low survival rate. Therefore, CHI3L1 has been proposed as a prognostic biomarker for neoplastic diseases [24].

In our exploration of EMP3 and CHI3L1, we discovered that both proteins are upregulated in malignant tumors such as glioma and are involved in tumor progression through the PI3K/AKT pathway. Furthermore, the two proteins may be linked to the tumor immune microenvironment and have been shown to regulate CD44. Previous studies have separately investigated the roles of EMP3 and CHI3L1 in gliomas. In this study, we analyzed the expression, prognosis, and survival curves of EMP3 and CHI3L1 in low-grade glioma and glioblastoma. Our findings suggest that the expression levels of both proteins may influence the prognosis of glioma patients, making them potential new prognostic markers.

## 2. Materials and Methods

### 2.1. GEPIA Database

GEPIA (http://gepia.cancer-pku.cn, accessed on 8 August 2022) is a newly developed interactive web server for analyzing the RNA sequencing and expression data of 9736 tumors and 8587 normal samples from The Cancer Genome Atlas (TCGA) and Genotype Tissue Expression (GTEx), using a standard processing pipeline [28]. We used the “Expression analysis Box Plots” module of the GEPIA2 (Gene Expression Profiling Interactive Analysis, version 2) web server (http://gepia2.cancer-pku.cn/#analysis, accessed on 8 August 2022) to obtain box plots of the expression differences between gliomas and normal brain tissues. We used the Kaplan–Meier “Survival Analysis” module of GEPIA2 to obtain the overall survival (OS) and disease-free survival (DFS) plot data of EMP3 and CHI3L1. 

### 2.2. CGGA Database

The CGGA database (http://www.cgga.org.cn, accessed on 10 August 2022) is a web application used for data storage and analysis [29]. It explored brain tumor datasets of over 2000 samples from Chinese cohorts. The clinicopathological data were downloaded from the CGGA database. We used the “Survival” module of CGGA to obtain survival plots of patients with primary and recurrent gliomas of different WHO grades. And the “Correlation” module was used to carry out Pearson correlation analysis.

### 2.3. cBioPortal

The cBio Cancer Genomics Portal (cBioPortal) (https://www.cbioportal.org, accessed on 10 August 2022) is a web site for exploring, visualizing, and analyzing multidimensional cancer genomics data [30,31]. We chose the “Merged Cohort of low-grade glioma (LGG) and GBM” in the “Quick select” section and entered “EMP3” and “CHI3L1” for queries on the genetic alteration characteristics of EMP3 and CHI3L1. Genetic alterations were observed in the “OncoPrint” module. We also used the “Comparison” module to obtain data on overall survival differences for glioma cases with or without EMP3 and CHI3L1 genetic alterations. Kaplan–Meier plots with log-rank P value were generated as well.

### 2.4. LinkedOmics

LinkedOmics (http://www.linkedomics.org, accessed on 12 August 2022) is a publicly available portal that includes multi-omics data from all 32 TCGA Cancer types [32]. To obtain a biological interpretation of the association results, genes co-expressed with EMP3 and CHI3L1 were displayed using the “LinkFinder” module. Enrichment analysis was performed on the “LinkInterpreter” module based on biological processes, cellular components, molecular functions, and network modules, among other functional categories.

### 2.5. RNA Extraction and Real-Time Quantitative PCR (qRT-PCR)

Total RNA was extracted from the cells using TRIzol (Invitrogen, Waltham, MA, USA) according to the manufacturer’s protocol. A RevertAid First Strand cDNA Synthesis Kit (K1622, Thermo Scientific^TM^, Waltham, MA, USA) was used for cDNA reversal. qRT-PCR was performed on the ABI StepOne^TM^ real-time Quantitative PCR instrument (Applied Biosystems, Waltham, MA, USA). CHI3L1 and EMP3 were standardized using GAPDH as the endogenous control. The primers used are listed in Table 1. The 2^−ΔΔCt^ values were used to compare the relative levels of target genes between the control and experimental groups. The test was performed in 3 replicates.

### 2.6. Samples

Glioma and normal tissues used in this study were obtained from the First Affiliated Hospital of Soochow University. The normal brain tissue used in our study was taken from the contusion brain tissue of patients undergoing surgery for traumatic brain injury, and all specimens were obtained with the informed consent of the patients or their families. The tumors were histopathologically classified according to WHO classification. Immediately after excision, the tissue samples were rapidly frozen in liquid nitrogen and stored at −80 degrees Celsius until further processing. All studies were approved by the Clinical Research Ethics Committee of Soochow University.

### 2.7. Statistical Analysis

We statistically analyzed the clinical characteristics of the 693 patients with CGGA. Variables with normal distribution were analyzed using the t-test; otherwise, the Mann–Whitney U test was used. A one-way analysis of variance was used for more than two data sets. Cox regression analysis was used to assess the prognostic value of the clinical factors based on data from the CGGA. Statistical analyses were performed using R (version 3.6.3). *p* values were two-sided, and a level of *p* value less than 0.05 was considered statistically significant. qRT-PCR analysis was performed using Graphpad Prism 8.0.2. A two-tailed Student’s *t*-test was used to analyze the statistical significance of the data compared between the two groups. The error bars represent the standard error (s.e.m.) of the measurement. *p* values are expressed by single asterisks (* < 0.05), double asterisks (** < 0.01), and triple asterisks (*** < 0.001). The number for each experiment is shown in the legend.

### 2.8. Immunohistochemistry (IHC)

The immunoreactivity (IR) of CHI3L1 and EMP3 was measured by immunohistochemistry, according to the standard procedure of immunohistochemistry, as well as the primary antibody of CHI3L1 (1:200, Proteintech) and EMP3 (1:200, Thermofisher, Waltham, MA, USA). GTVisionTM III Detection System/Mo&Rb (including DAB) secondary antibody kit was used (Genetech, Shanghai, China). Finally, the sections were reverse-stained with hematoxylin (KeyGEN BioTECH, Nanjing, China). All images were taken using an ECLIPSE Ti2 microscope (Nikon, Japan).

## 3. Results

### 3.1. The Expression Levels of EMP3 and CHI3L1 in Glioma Were Higher Than in Normal Brain Tissues

**Figure 1:**
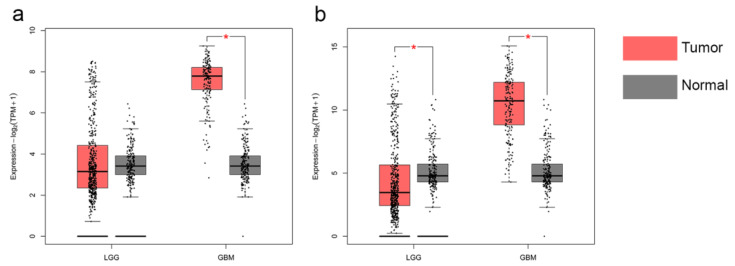
Transcriptional levels of EMP3 (**a**) and CHI3L1 (**b**) in normal brain tissue and glioma.

To investigate the expression levels of EMP3 and CHI3L1 in different tissues, we analyzed the mRNA levels of EMP3 and CHI3L1 in normal brain tissue, low-grade gliomas, and high-grade gliomas. We included mRNA data from 888 patients, from the GEPIA database, including 518 patients with low-grade gliomas, 207 patients with high-grade gliomas, and 163 normal patients. We found that the expression levels of EMP3 and CHI3L1 were higher in low-grade and high-grade gliomas than in normal brain tissue, and that the levels of EMP3 and CHI3L1 were much higher in high-grade gliomas than in normal brain tissue (Figure 1).

### 3.2. Clinical Characteristics of Glioma Patients

We found relevant data from 693 patients with glioma in the CGGA database and classified them according to age, gender, histology, tumor type, isocitrate dehydrogenase (IDH) mutation level, 1p19q level, and O-6-methylguanine DNA methyltransferase (MGMT) level. The expression levels of EMP3 and CHI3L1 were calculated, and the correlation was verified by an independent sample t-test using the R (version 3.6.3) software.

As shown in Table 2, the expression levels of EMP3 and CHI3L1 were higher in patients aged 45 years or older than in those aged under 45 years. The expression levels of EMP3 and CHI3L1 were higher in female patients. The expression levels of EMP3 and CHI3L1 in GBM patients were higher than those in non-GBM patients. The expression levels of EMP3 and CHI3L1 increased with an increase in the WHO grade. The expression levels of EMP3 and CHI3L1 in wild-type IDH-1, non-1P19Q co-deletion, and MGMT non-methylated gliomas were higher than in others. In conclusion, the average expression levels of EMP3 and CHI3L1 were statistically significant among the eight clinical characteristics of glioma patients.

### 3.3. The Prognostic Significance of the Expression Levels of EMP3 and CHI3L1 in Glioma

**Figure 2:**
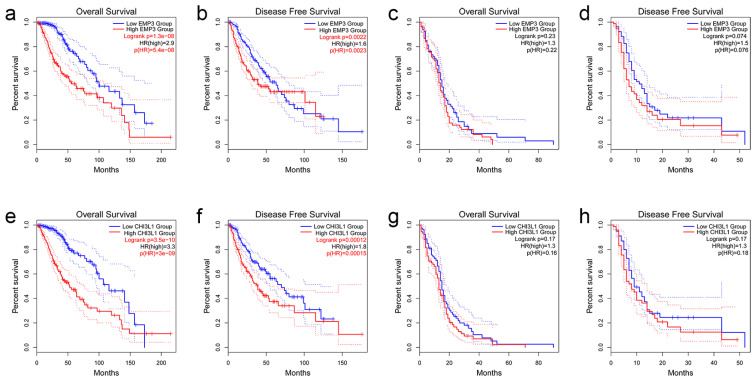
OS and DFS analyses based on EMP3 and CHI3L1 expression in LGG (**a**,**b**,**e**,**f**) and GBM (**c**,**d**,**g**,**h**) using the TCGA database.

To understand the effect of EMP3 expression level on patient survival, 514 patients with low-grade glioma and 162 patients with GBM were selected from the TCGA database, and patients with LGG and GBM were divided into two groups according to the median expression level of EMP3. The OS and DFS time curves were described. Prognosis showed that LGG patients with low EMP3 expression had prolonged survival and disease-free survival compared to LGG patients with high EMP3 expression (Figure 2a,b). Meanwhile, in GBM patients, the survival time and disease-free survival time of GBM patients with low EMP3 expression levels were not significantly different from those of GBM patients with high EMP3 expression levels, and no statistical significance was found after the *t*-test (Figure 2c,d).

Similarly, the survival and disease-free survival curves of LGG and GBM patients with high and low CHI3L1 levels were plotted according to the median level of CHI3L1 expression. Prognosis showed that LGG patients with common CHI3L1 expression had longer disease-free survival, similar to EMP3 (Figure 2e,f). The expression level of CHI3L1 did not significantly affect the survival time or disease-free survival time patients (Figure 2g,h).

In conclusion, EMP3 and CHI3L1 can be used as prognostic indicators in low-grade gliomas, and the OS and DFS of patients with low EMP3 and CHI3L1 are significantly better than those of patients with high EMP3 and CHI3L1. However, the expression levels of EMP3 and CHI3L1 had no significant effect on the prognosis of GBM patients.

### 3.4. Prognostic Significance of EMP3 and CHI3L1 in Primary and Recurrent Gliomas of CGGA

**Figure 3:**
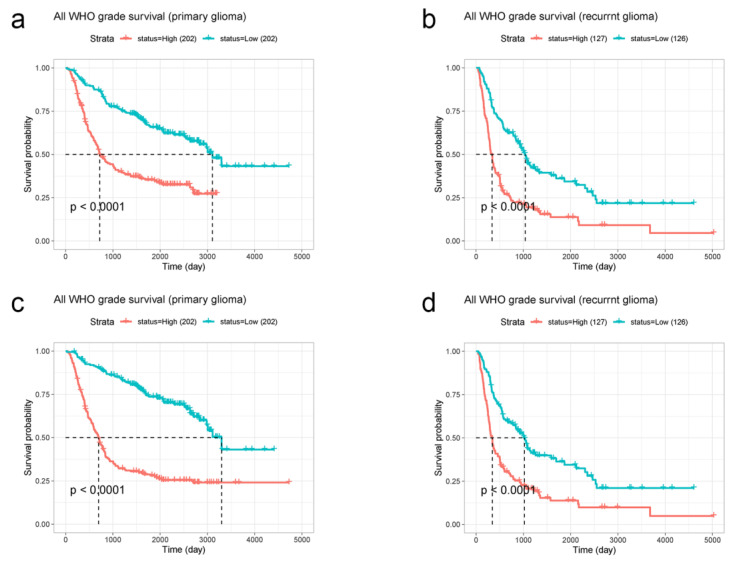
CGGA database was used to analyze the relationship between EMP3 (**a**,**b**) and CHI3L1 (**c**,**d**) expression levels and the survival time of primary and recurrent gliomas.

We selected 404 primary glioma patients and 253 recurrent glioma patients from the CGGA database, including all glioma types. Similarly, EMP3 and CHI3L1 were classified according to their median expression levels. A survival curve was drawn according to the survival time, and the median survival was statistically analyzed. Survival curves showed that in primary and recurrent gliomas, the survival time of patients with low levels of EMP3 was superior to that of patients with high levels of EMP3, with *p* < 0.001 (Figure 3a,b). The survival curve of CHI3L1 was similar to that of EMP3 (Figure 3c,d). 

According to these results, the expression levels of EMP3 and CHI3L1 are significant in the prognostic prediction of both primary and recurrent gliomas. Patients with tumor cells with low EMP3 and CHI3L1 expression levels had a better prognosis than patients with tumor cells with high EMP3 and CHI3L1 expression levels.

### 3.5. COX Regression Analysis of Overall Survival of Glioma Patients

**Figure 4:**
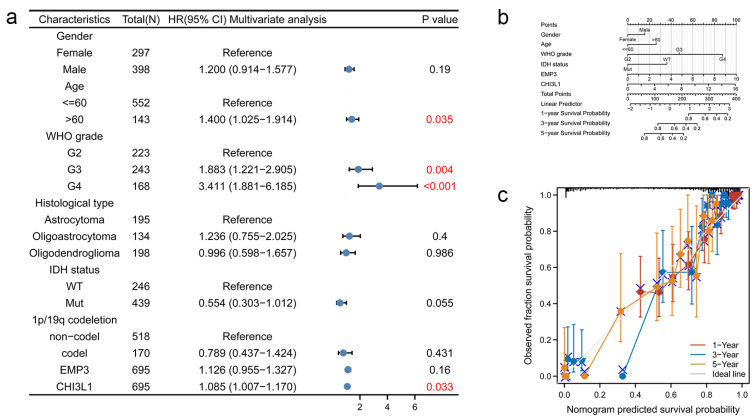
Nomogram development and validation. (**a**) For OS, hazard ratios and *p*-value of constituents involved in multivariate Cox regression considering clinical information and prognostic EMP3 and CHI3L1 in TCGA database. The numbers in red represent *p* < 0.05; (**b**) the Nomogram graph of the Cox survival curve regression analysis model; (**c**) Nomogram calibration curve for Nomogram OS prognostic model.

Different ages, sex, tumor type, and various genetic phenotypes can affect the survival of glioma patients. In order to explore the overall survival of glioma patients, 695 glioma patients were selected from the TCGA database, and eight factors including age and gender were analyzed using a COX multivariate regression analysis. The cut-off value was *p* < 0.05, and the results were visually altered. The 95% confidence interval (CI) of gender, pathological type, IDH status, 1p/19q deletion, and EMP3 expression level all crossed one, and these five factors had no significant impact on the OS of glioma patients (*p* > 0.05). The 95% CI of age, WHO grade, and CHI3L1 were all on the right side of one (*p* < 0.05), and these three factors affected the overall survival of glioma patients (Figure 4a).

We used a multivariate Cox regression analysis model to more directly reflect and predict the survival rate of glioma patients. Scoring items were age, gender, WHO level, IDH status, EMP3, and CHI3L1 (Figure 4b). The Nomogram OS prognostic model was calibrated (Figure 4c).

### 3.6. Gene Changes and Prognosis of EMP3 and CHI3L1 in Glioma

**Figure 5:**
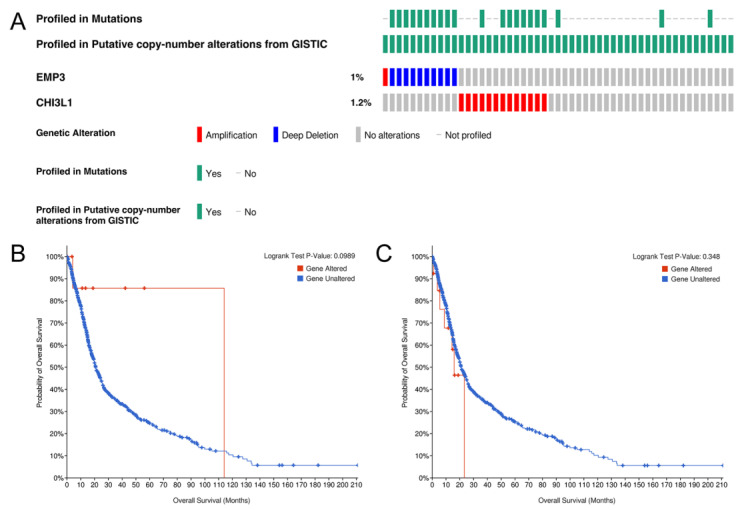
Genetic alteration of EMP3 and CHI3L1 (**A**) and the association with OS in gliomas (**B**,**C**).

Based on the TCGA database, we analyzed the influence of gene changes affecting EMP3 and CHI3L1 expression in glioma patients on the survival time of glioma patients. We pooled and analyzed the genetic information of 693 glioma patient samples from the CGGA database (Figure 5A) and found that the mutation rates of EMP3 and CHI3L1 were 1% and 1.2% in 693 patients.

To further understand the influence of mutations in the EMP3 and CHI3L1 genes on the prognosis of glioma patients, we performed a predictive analysis and a *t*-test, respectively. The results showed that in EMP3 and CHI3L1, gene mutations had no significant effect on the survival prognosis (Figure 5B,C).

**Figure 6:**
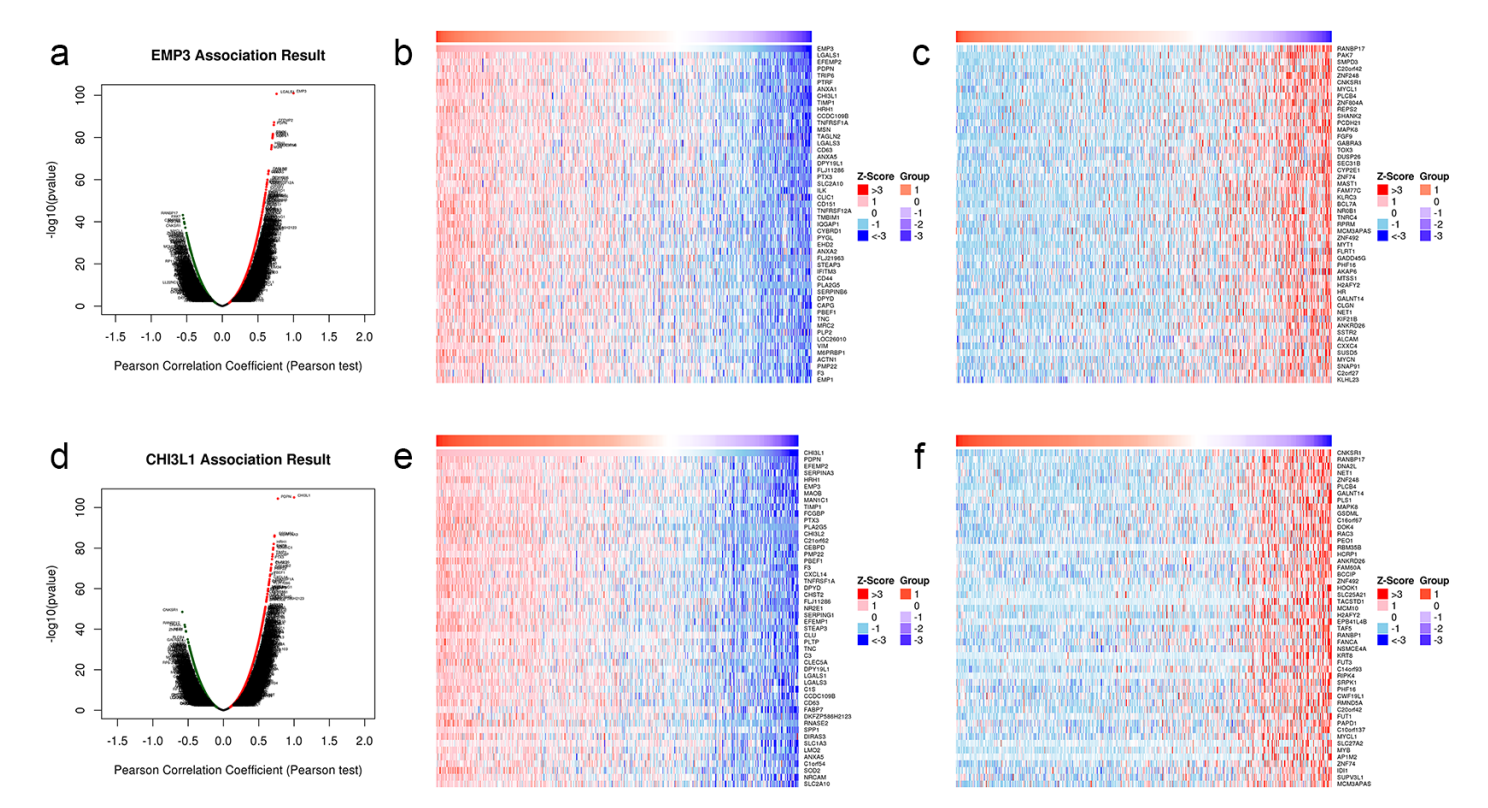
Co-expression genes and heat map of EMP3 (**a**–**c**) and CHI3L1 (**d**–**f**) in glioma cells.

To understand the co-expression genes of EMP3 and CHI3L1 regulation in glioma, we analyzed the conditions of EMP3 and CHI3L1-related genes (Figure 6a,b). The heat map is drawn according to its related situation (Figure 6c–f). 

### 3.7. There Is Correlation between EMP3 and CHI3L1

It can be seen from the above that there are co-expressed genes between EMP3 and CHI3L1 in glioma, and they have similar effects on the prognosis of glioma patients. We further verified the correlation between them.

**Figure 7:**
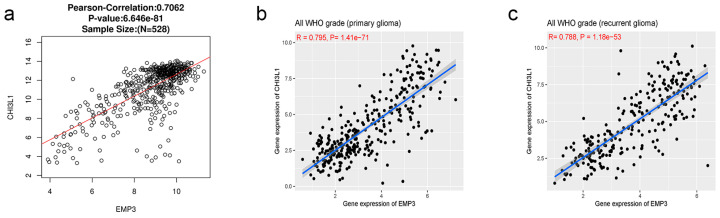
Genetic correlation of EMP3 and CHI3L1 with Pearson correlation analysis in Linkedomic (**a**) and CGGA databases (**b**,**c**).

A correlation analysis of these two proteins was conducted in 528 normal human (Figure 7a), and the correlation analysis showed a Pearson correlation of 0.706 (*p* < 0.001), indicating a specific positive correlation between the two proteins in normal human subjects.

To further verify the correlation between EMP3 and CHI3L1 in patients with glioma, we analyzed the correlation between these two proteins in patients with primary and recurrent gliomas. The results showed that EMP3 positively correlated with CHI3L1 in both primary and recurrent gliomas (Figure 7b,c).

### 3.8. GO and KEGG Pathway Enrichment Analysis of EMP3 and CHI3L1 Co-Expression Genes in Glioma

**Figure 8:**
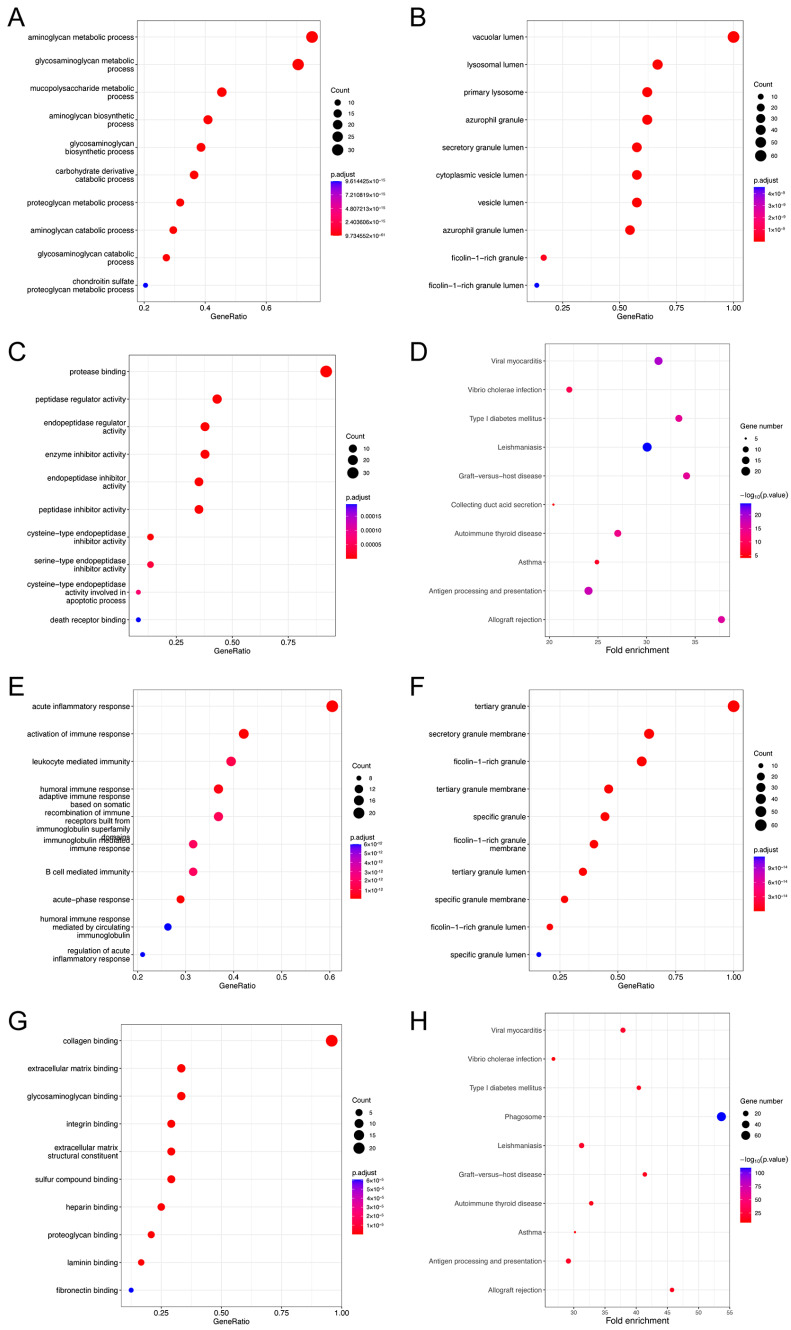
Gene set enrichment analyses of EMP3 and CHI3L1 co-expression genes in gliomas. (**A**–**C**) BP, CC, and MF of EMP3 co-expression genes. (**D**) Enrichment analysis of EMP3 by KEGG pathway. (**E**–**G**) BP, CC, and MF of CHI3L1 co-expression genes. (**H**) Enrichment analysis of KEGG pathway in CHI3L1. Only *p* < 0.05 disease was present.

We performed a GO analysis and a KEGG analysis for EMP3 and CHI3L1, respectively. The GO analysis showed that the biological process (BP) of EMP3 was mainly related to the metabolism of aminoglycan metabolic process and glycosaminoglycan metabolic processes (Figure 8A); and the cellular components (CCs) were concentrated primarily in the vacuolar lumen and lysosomal lumen (Figure 8B). The molecular function (MF) of EMP3 was significantly enriched in protease binding (Figure 8C). The KEGG enrichment analysis showed that EMP3 was significantly correlated with viral myocarditis and leishmaniasis (Figure 8D). The BP of CHI3L1 was mainly related to acute inflammatory response and immune response activation (Figure 8E). The CCs were primarily concentrated in the tertiary granule and secretory granule membrane (Figure 8F), and the MF was enriched in collagen binding (Figure 8G). The KEGG enrichment analysis revealed a significant relationship between CHI3L1 and phagosomes (Figure 8H).

### 3.9. The Expressions of CHI3L1 and EMP3 Were Low in Glioma Cells and Decreased with an Increase in Glioma Grade

**Figure 9:**
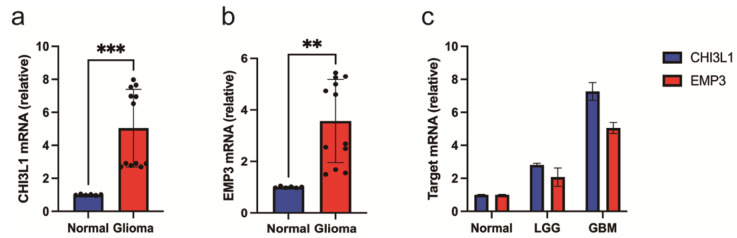
**qRT-PCR results of tissue samples.** (**a**–**c**) CHI3L1 and EMP3 levels in normal and glioma cells were determined by qRT-PCR. (** *p*< 0.01, and *** *p* < 0.001).

To validate our analysis, we measured the expression of CHI3L1 and EMP3 in normal and low-grade glioma cells and glioblastomas. The results of qRT-PCR showed that both CHI3L1 and EMP3 were expressed at low levels in tumor tissues (Figure 9a,b), and the expression levels decreased with an increase in the glioma grade (Figure 9c). This is consistent with the results of the bioinformatics analysis. 

### 3.10. IHC Results of CHI3L1 and EMP3 in Patient Specimens

**Figure 10:**
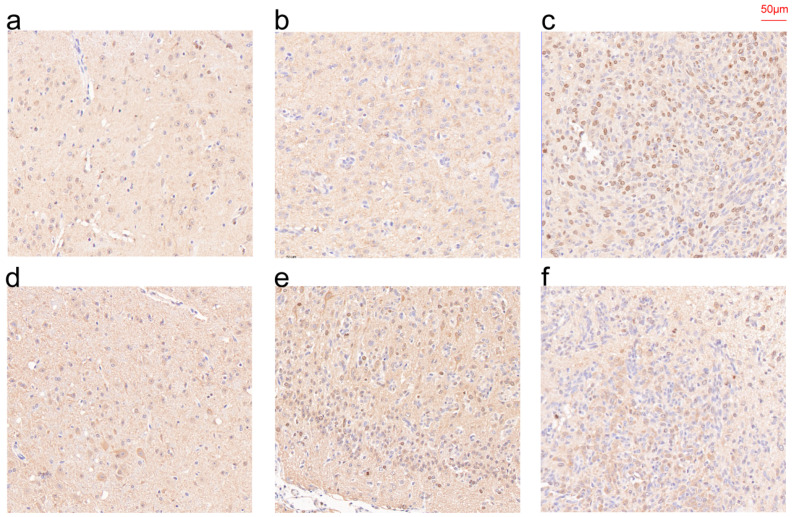
IHC of CHI3L1 (**a**–**c**) and EMP3 (**d**–**f**) in normal tissue (**a**,**d**), LGG (**b**,**e**), and GBM (**c**,**f**).

Immunohistochemical techniques were employed to measure the expression of CHI3L1 and EMP3 in paraffin sections of normal brain tissue, LGG and GBM (Figure 10). The results of our statistical analysis and qRT-PCR were corroborated by the findings that the expression of CHI3L1 and EMP3 increased with the rise in tissue malignity.

## 4. Discussion

In this study, using biological information analysis, we obtained the relevant expression levels of EMP3 and CHI3L1 and the patient phase of TCGA and CGGA. We analyzed the survival time, disease-free survival time, gene mutations, and the correlation between them in various gliomas.

The results indicate that a low expression of EMP3 and CHI3L1 significantly affects patients with low-grade gliomas, showing a better prognosis. This conclusion is consistent with the analysis of Zhao T [24]. In addition, we analyzed the gene expression of EMP3 and CHI3L1. The results showed that EMP3 and CHI3L1 had a specific correlation with the prediction of glioma and could be used as a combined index to judge the prognosis of glioma patients. 

Previous studies [9,20,21,22,23,33] have shown that EMP3 and CHI3L1 are upregulated in glioma tissues. Using available survival data from expression profiles and TCGA data sets, a Kaplan–Meier analysis showed that these two genes were associated with glioma survival, including grade, age, and treatment [34].

Although EMP3 was initially identified as a tumor suppressor in low-grade gliomas, its inhibitory effect remains controversial [20,21]. CHI3L1, a gene encoding YKL-40, is a marker of the glioblastoma mesenchymal subtype and is overexpressed in glioblastoma tumor stem cells [35]. In GBM, EMP3 expression is upregulated to promote the progression of brain tumors, and a high EMP3 expression is associated with poor prognosis [36,37]. Similarly, in gliomas, CHI3L1 expression is associated with glioma prognosis [38], and the prognosis of glioma patients with high CHI3L1 expression is poor [39]. Our study confirmed a correlation between EMP3 and CHI3L1 in glioma cells.

Our analysis showed that the expression levels of EMP3 and CHI3L1 in GBM had no significant impact on the OS of patients with GBM. Patients with low-grade gliomas with low EMP3 and CHI3L1 expression levels had significantly better OS. This is consistent with the results of Scrideli [20] and Bolin [21]. Deluche et al.’s study on CHI3L1 also showed that patients with low-grade glioma with low CHI3L1 expression had a better quality of life [37]. Reflections on all-grade glioma patients showed that EMP3 [21] and CHI3L1 [37] showed significant differences in the expression of low-grade and high-grade gliomas. Therefore, we believe that the expression level of EMP3 and CHI3L1 have guiding significance for the prognosis and survival of patients with low-grade glioma. However, although the expression level of EMP3 and CHI3L1 in GBM is higher than that in normal brain tissue, their significance for the prognosis of GBM patients remains to be confirmed.

However, the mechanisms of action of EMP3 and CHI3L1 are still under investigation. It has been reported that EMP3 directly interacts with TGFBR2 in glioma cells. This subsequently activates the TGF-β/Smad 2/3 pathway and enhances tumor progression in vitro and in vivo [22]. Chen Q et al. found that EMP3 is involved in the immunosuppression of GBM, and EMP3 knockout inhibited tumorigenesis and produced survival advantages in mice [39]. EMP3 knockdown decreased the levels of p-AKT, p-ERK as well as p-EGFR, and weakened cell proliferation, suggesting that EMP3 regulates receptor tyrosine kinase-mediated mitotic signaling [13]. The GO analysis showed that the components of CHI3L1 cells were mainly concentrated in the tertiary granule and granular secretory membrane, and promoter methylation of CHI3L1 resulted in the loss of mesenchymal characteristics in these cells [40]. Therefore, it was proposed that there might be a correlation between CHI3L1 expression and IDH mutations by inducing methylation of the CHI3L1 promoter [41]. The hypermethylated phenotype of CHI3L1 may also lead to low CHI3L1 expression and favorable prognosis [40,41].

CD44, a cell surface transmembrane glycoprotein, is considered a key signaling regulator of cell growth, survival, and differentiation [42]. Martija et al. showed that EMP3 regulates erythropoiesis by stabilizing and organizing the distribution of CD44 in the plasma membrane [43]. Similarly, Thornton et al. demonstrated a functional association between EMP3 and CD44 in red blood cells [44]. Jun et al. demonstrated the ability of EMP3 to regulate TGF-β signaling using a set of glioblastoma cell lines rich in the MES GBM marker CD44 [22]. CHI3L1 plays a role in inflammation and tissue repair by regulating TGF-β expression [45]. Ponta H et al. reported that CHI3L1 physically interacts with CD44 to activate the ERK and Akt pathways [42]. Hsieh et al. studied hepatocellular carcinoma cells and found that Akt overexpression reduced HCC cell migration, suggesting that the invasion-promoting function of EMP3 is mainly mediated by the PI3K/Akt pathway [16]. EMP3 knockdown decreased total ERK and phosphorylated ERK (p-ERK) [43]. Therefore, EMP3 and CHI3L1 may affect the proliferation, apoptosis, and death of glioma cells by regulating CD44 and affecting TGF-β, ERK, Akt, and other pathways.

In previous studies, EMP3 [8] and CHI3L1 [24] were identified as novel independent predictors of clinical diagnosis, prognosis, and immune infiltration in glioma patients, respectively. In our study, we combined EMP3 and CHI3L1, which are correlated in glioma cells and can be combined as prognostic factors for patients with low-grade glioma. Based on these conclusions, we speculated that it could be combined with the IDH gene to predict the prognosis of glioma patients. Although mutations in IDH are a predisposing event in glioma development, recent genome-wide mutation analyses have demonstrated that IDH mutations are prevalent in more than 70% of WHO Grade II and III gliomas. In comparison, less than 5% of major GBMs have these mutations [46]. 

In this study, we have concluded that low expression of EMP3 and CHI3L1 represents a better prognosis, so can we artificially inhibit the expression of EMP3 and CHI3L1? Lange et al. predicted that conventional therapy (radiotherapy and temozolomide) combined with CHI3L1 targeted inhibitors, mainly grade IV chemotherapy, may provide better outcomes for patients with a poor prognosis (IDH wild-type, no 1p19q co-deletion, high CHI3L1 expression, and low NTRK2 expression) in the follow-up treatment of glioma [33]. We speculate that targeted inhibitors of EMP3 and CHI3L1 genes may be an effective method to improve the prognosis of glioma patients in the future.

The limitations of this study are as follows: First of all, we only used TCGA and CGGA databases to analyze 693 patient samples, with a small sample size and possible bias. Furthermore, gliomas were simply classified by the WHO classification in terms of 2/3/4 grade without refining glioma types, such as molecular typing and IDH mutation. In addition, in this paper, we only studied the relationship between the expression levels of EMP3 and CHI3L1 and the prognosis of glioma patients, without studying the specific pathway, which is also the focus of our research in the next stage. Moreover, in this study, we conducted statistical analyses on the role of EMP3 and CHI3L1 in glioma from existing public data and verified the results with a simple qRT-PCR validation in human specimens. Subsequent research will involve regulating the expression of these two genes through siRNA and plasmid, exploring relevant molecular processes and signaling pathways in cell lines and animal models. We will also collect serum and cerebrospinal fluid samples from clinical patients to measure EMP3 and CHI3L1 expression levels. Additionally, we are planning to set up a multicenter study to investigate the correlation between EMP3 and CHI3L1 expression levels and survival outcomes in glioma patients.

## 5. Conclusions

We used TCGA and CGGA databases to analyze the effect of EMP3 and CHI3L1 expression on the prognosis of glioma patients and their correlation with gene expression using bioinformation analysis. The results showed that low-grade glioma patients with low expression of EMP3 and CHI3L1 had better prognosis, and EMP3 and CHI3L1 co-expression genes were correlated. The combination of these two factors can be a new prognostic index of glioma patients.

## Figures and Tables

**Table 1:** Primers for qRT-PCR.

Genes	Primers (5′-3′)
EMP3-forward	AAGATCAGTACCTCTCAGATGG
EMP3-reverse	GCAGCACAAGAGACGTATCATA
CHI3L1-forward	CTGTGGGGATAGTGAGGCAT
CHI3L1-reverse	CTTGCCAAAATGGTGTCCTT
Human-GAPDH-forward	GGAAGCTTGTCATCAATGGAAATC
Human-GAPDH-reverse	TGATGACCCTTTTGGCTCCC

**Table 2:** Eight clinical characteristics of glioma patients.

		Case (*N*)	Expression of CHI3L1	*p* Value	EMP3 Expression	*p* Value
Age	<45	383	351.93 ± 56.01	<0.001	53.98 ± 4.48	<0.001
	>=45	310	1242.16 ± 167.98		122.92 ± 10.46	
Gender	Male	398	768.95 ± 114.63	<0.001	92.49 ± 7.97	<0.001
	Female	295	724.80 ± 118.66		74.47 ± 6.89	
Histology	LGG	444	247.71 ± 56.08	<0.001	39.12 ± 3.41	<0.001
	GBM	249	1646.08 ± 195.80		166.32 ± 12.31	
WHO	II	188	170.43 ± 43.25	<0.001	36.31 ± 5.75	<0.001
	III	256	304.46 ± 91.85		41.18 ± 4.14	
	IV	249	1646.08 ± 195.80		166.32 ± 12.31	
Tumor type	Primary	422	596.04 ± 90.95	<0.001	80.28 ± 7.73	<0.001
	Recurrence	271	990.14 ± 156.96		91.89 ± 6.99	
IDH-1 status	Mutant	356	157.87 ± 53.79	<0.001	25.24 ± 2.56	<0.001
	Wild-type	286	1588.96 ± 177.28		154.13 ± 9.85	
1p19q status	Codeletion	145	66.72 ± 29.58	<0.05	23.93 ± 5.34	<0.001
	Non-codeletion	478	1049.18 ± 117.30		114.40 ± 7.33	
MGMT status	Methylation	315	608.28 ± 93.16	<0.001	84.80 ± 8.98	<0.001
	Non-methylation	227	894.40 ± 154.03		102.02 ± 9.66	

## Data Availability

The data analyzed in the current study are available in the Oncomine, GEPIA, LinkedOmics, cBioPortal and CGGA databases.
